# Local anaesthesia vs. brachial plexus block in trapeziometacarpal joint arthroplasty

**DOI:** 10.1007/s00402-024-05637-3

**Published:** 2024-12-21

**Authors:** Maximilian Moshammer, Sebastian Martin Klim, Reingard Glehr, Georg Hauer, Andrzej Hecker, Andreas Leithner, Mathias Glehr

**Affiliations:** 1 Department of Orthopaedics and Trauma, Medical University of Graz, Auenbruggerplatz 5, Graz, 8036 Austria; 2 Division of Plastic, Aesthetic and Reconstructive Surgery, Department of Surgery, Medical University of Graz, Auenbruggerplatz 29 34 /4, Graz, 8036 Austria; 3 COREMED – Centre for Regenerative Medicine and Precisions Medicine, Joanneum Research Forschungsgesellschaft mbH, Neue Stiftingtalstrasse 2, Graz, 8010 Austria; 4 Institute of General Practice and Evidence-based Health Services Research, Medical University of Graz, Auenbruggerplatz 20, Graz, 8036 Austria

**Keywords:** Trapeziometacarpal arthroplasty, Local anaesthesia, Pain, Moovis prosthesis, Reliability, Anxiety

## Abstract

**Introduction:**

An established anaesthetic procedure used during total trapeziometacarpal joint (TMCJ) arthroplasty is the brachial plexus block (BPB). It was hypothesized that local anaesthesia (LA) provides advantages in overall cost, enables intraoperative assessment of the prosthesis, and minimises the anaesthetic risk. In this study, LA to BPB was compared and outcomes, safety, and overall patient satisfaction were analyzed.

**Materials and methods:**

In this single-center cohort study, 32 patients (34 operated thumbs) who underwent total TMCJ arthroplasty between February 2018 and July 2021 were included. Two groups were formed depending on the anaesthetic method used. One group was operated under LA, and the other under BPB. Functionality scores were assessed preoperatively and three month postoperatively. Additionally, pain was assessed on the 1st and 12th postoperative day. Data on intraoperative pain and anxiety, overall satisfaction, pain medication use, and willingness to undergo the procedure again were gathered through a retrospective telephone survey.

**Results:**

No significant differences between LA and BPB were found in terms of functional outcomes, pain reduction, and willingness to repeat the procedure. The analysis further showed significant differences in intraoperative anxiety (higher in the LA group; LA 1.69, SD: 2.65; BPB 0.28, SD: 0.58; measured using a numeric rating scale 0–10; *p* = 0.045), operation length (higher in BPB group; LA 39 min, SD: 7.46; BPB 45 min, SD: 7.02; *p* = 0.018) and overall setup time (higher in BPB group; LA 76 min, SD: 15.85; BPB 102 min, SD: 19.66; *p* < 0.001). No conversion from LA to another anaesthetic method was necessary.

**Conclusion:**

The use of LA in total TMCJ arthroplasty is a practical and reliable alternative to the well-established BPB. LA reduces the cost of the procedure, necessity of an anaesthesiology team and the duration of the patients´ hospital stay. Patients should be actively involved in selecting the anaesthetic method to optimize the operative procedure and overall outcome.

## Introduction

Trapeziometacarpal joint (TMCJ) arthritis is a highly prevalent condition. Osteoarthritis (OA) of the TMCJ has the second highest prevalence of OA in the hand after the distal interphalangeal joint [1, 2]. Several studies have confirmed a correlation between age, sex, and the prevalence of TMCJ arthritis [3–7]. Compared to the conventional resection-suspension-arthroplasty, total TMCJ arthroplasty offers an alternative treatment by replacing the joint with an artificial joint, thus preserving the joint’s geometry. In this trial, the MOOVIS^®^ prosthesis (Stryker, Kalamazoo, Michigan, USA) was utilized [8]. In many countries, the availability of anaesthesiology teams is limited, making the ability to perform procedures with fewer resources increasingly important [9]. While the brachial plexus block (BPB) is an established anaesthetic method in hand surgery [10], there is limited data concerning the safety of local anaesthesia (LA) in total TMCJ arthroplasties. Only a few studies have been published comparing LA with BPB or general anaesthesia in total TMCJ arthroplasty. One case report, a retrospective review of cases and two comparative studies were found [11–14]. However, none of these studies compared the intraoperative anxiety experienced by patients undergoing total TMCJ arthroplasty with LA versus BPB. Possible advantages of LA compared with BPB include minimized anaesthetic risk, overall resource savings, the potential for outpatient treatment, and the ability to perform intraoperative assessments of active stability and range of motion of the prosthesis [13].

The aim of this study was to investigate the impact of LA versus BPB in total TMCJ arthroplasty, focusing on pain during and after the procedure, functional outcomes, length of the operation, and overall satisfaction. In contrast to the studies mentioned above, the aim was also to compare the intraoperative anxiety experienced by patients. The hypothesis is that LA is equivalent to BPB in terms of pain reduction, intraoperative anxiety, functional outcomes, and overall satisfaction.

## Materials and methods

### Patients

From February 2018 to July 2021, 40 patients were treated with a TMCJ prosthesis in a tertiary referral center for hand surgery. Two patients underwent surgery on both hands, resulting in 42 operated thumbs. Inclusion criteria for this study were: patients diagnosed with TMCJ arthritis with no degenerative changes in the scaphotrapeziotrapezoid joint who underwent a total TMCJ arthroplasty and who were operated on using either LA or BPB. No patients operated under general anaesthesia were included in this study. In all cases, conservative treatment options had been exhausted. Informed consent was provided by every patient.

### Study design

The data for this monocentric study was collected prospectively from a study comparing total TMCJ arthroplasty with Epping-arthroplasty [15], combined with a retrospective data supplementation to gather more information about the study question. In this study, only patients that received surgical treatment with a TMCJ prosthesis were considered for inclusion. One group received LA and the control group received BPB. Patients operated on under LA were outpatients, whereas those operated on under BPB were inpatients. The patients could decide preoperatively which anaesthetic technique they preferred. The medical team that performed the clinical examinations perioperatively was blinded regarding the applied anaesthetic procedure. The clinical examinations were complemented by a telephone survey. All operations were performed by the same surgeon.

### Data collection

The collected data included the DASH-score, the VAS (visual analogue scale for pain) and the McGill Pain Questionnaire [16, 17].The examinations were performed preoperatively and three months postoperatively. The VAS was additionally evaluated one the 1st postoperative day and the 12th postoperative day (suture removal).

Data regarding operation length and setup time were recorded. Operation time was defined as the cut-seam time, whereas the setup time was defined as the period from the patient’s first contact with medical personnel in the operating theatre until the patient left the operating theatre.

A telephone survey was further conducted with a mean time of 26 months after surgery. A questionnaire was developed that provided consistent and clear answers to allow for proper interpretation. The questionnaire was developed in a team of three scientific experts and tested on a cohort of ten randomly chosen sample patients. Information regarding the patients’ pain during the operation, anxiety during the operation, and overall satisfaction was gathered with this questionnaire. Patients could choose between 0 and 10 points to answer each question. Depending on the subject asked, zero points meant no pain, no anxiety, and not at all satisfied, while ten points meant the worst pain imaginable, the strongest anxiety, and being completely satisfied. Information regarding the sufficiency of the basic pain medication and willingness for reoperation with the same anaesthetic method was also gathered using this questionnaire, where patients could answer “yes” or “no”.

### Method of operation

**Fig. 1:**
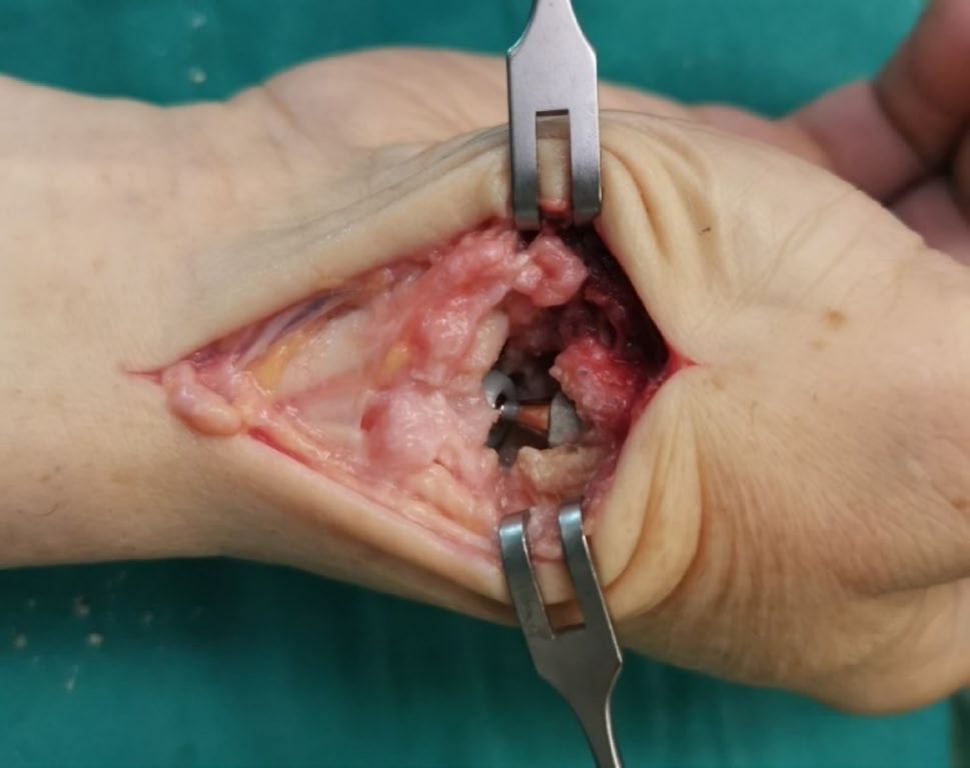
In-situ trapeziometacarpal joint prosthesis

The performed operation in this study was a replacement of the TMCJ with the Moovis^®^ prosthesis (Fig.1). To address the issues of cup loosening or luxation, this prosthesis features a dual mobility cup. Several cohort studies have investigated the Moovis^®^ prosthesis [18–20], and the prosthesis had satisfactory results with low failure, loosening, and dislocation rates when compared to other prosthesis used [21]. All operations were performed by the same surgeon at the same hospital.

After application of a tourniquet, a dorsolateral approach along the axis of the TMCJ was used. The superficial branch of the radial nerve was identified and the tendons of the abductor pollicis longus and opponens pollicis were preserved. The joint capsule was split longitudinally, a synovectomy performed, and osteophytes excised. Between 2 and 3 mm of the base of the first metacarpal was resected, and the medullary canal of the first metacarpal was opened and enlarged using progressively larger rasps. The stem was then inserted, after which a k-wire was anchored into the trapezium and the shape of the cup was milled into the trapezium. Using a trial head and neck, the patient was then asked to rotate the thumb to ensure appropriate stability, range of motion, and length of the thumb. In the BPB group, these assessments were performed passively. Afterwards the definitive head and neck implants were inserted. After wound closure, the thumb was immobilized for 6 weeks with a splint. After the 5th week, the patients commenced physiotherapy.

### Method of anaesthesia

The TMCJ is innervated by the median nerve, the radial nerve, the lateral cutaneous nerve of the forearm and the ulnar nerve. The branches from the median nerve arise from the thenar branch of the median nerve, the palmar branch, and sometimes directly from the median nerve in the carpal tunnel. The branches from the radial nerve arise from the superficial radial nerve. The lateral cutaneous nerve of the forearm innervates the TMCJ via Cruveilhier’s branch. The TMCJ is also innervated by the deep branch of the ulnar nerve. Of all sensory branches, the median nerves dominate in number and calibre [22–27]. With this anaesthetic method, all possible branches leading to the TMCJ are expected to be numbed.

**Fig. 2:**
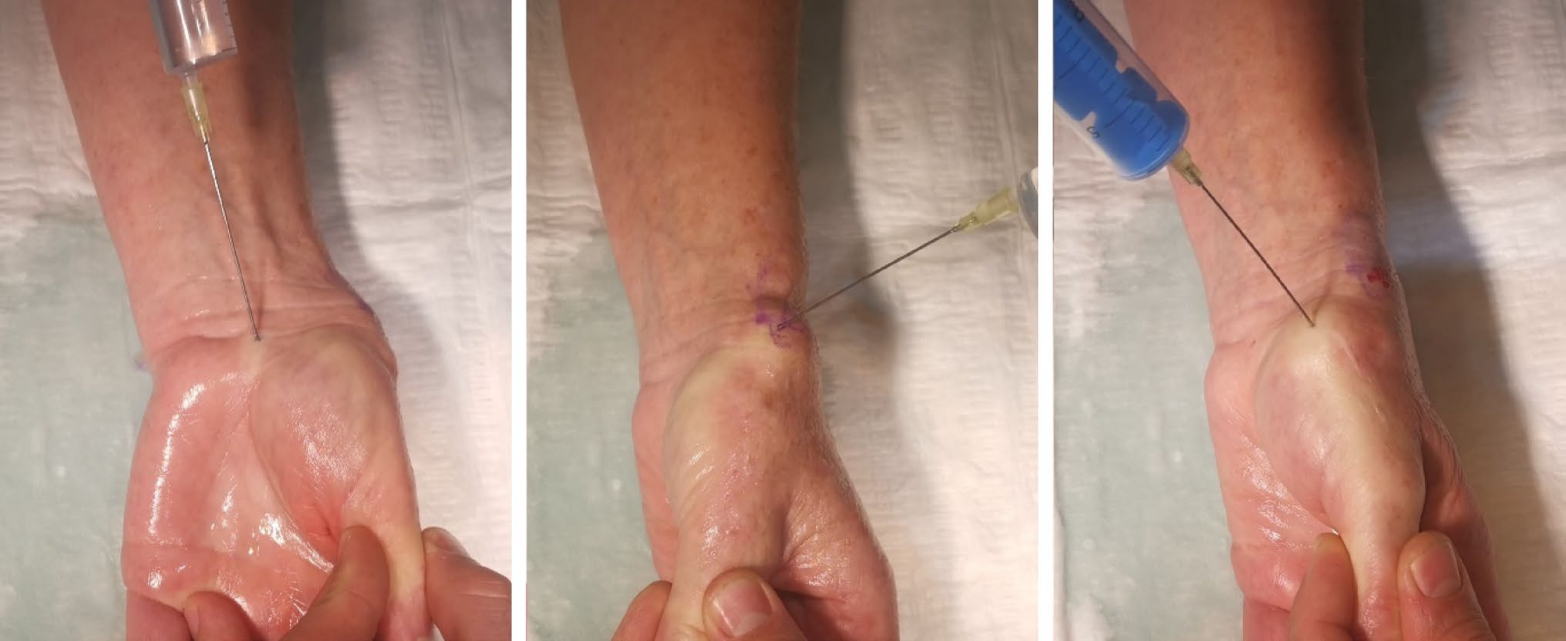
Application of the LA: from left to right, median nerve block; superficial radial nerve block; intraarticular injection

The LA solution used was Xyloneural^®^ (Pharmore GmbH, Ibbenbüren, Germany) 1%. 1 ml of this solution contains 10.7 mg of lidocaine hydrochloride 1 H_2_O. For this procedure, 20 ml of the solution was prepared. The surgeon first supinated the hand and started with a block of the median nerve by injecting 5 ml of the solution into the carpal tunnel. The hand was then pronated 45 degrees and a block of the superficial radial nerve was carried out by infiltrating 5 ml of the solution at the proximal aspect of the tabatière anatomique. The last step involved injecting 3 ml of the solution directly into the joint space of the TMCJ. The surgeon then waited 20 min for the solution to properly disperse into the tissue (Fig. 2).

The anaesthetics used for the BPB in this study were Mepinaest^®^ 1.5% (Gebro Holding GmbH, Fieberbrunn, Austria; a mixture of Mepinaest^®^ 1% and Mepinaest^®^ 2%; active compound: Mepivacain) and Ropinaest^®^ 7.5 mg/ml (Gebro Holding GmbH, Fieberbrunn, Austria; active compound: Ropivacain).

No tranquilizers were administered preoperatively with either anaesthetic method. Postoperatively, each inpatient and outpatient received one infusion of 75 mg Diclofenac and 30 mg Orphenadrine. The subsequent oral pain medication consisted of 50 mg Diclofenac 1-0-1, 20 mg Pantoprazole 1-0-0 during NSAID intake, and 500 mg Metamizole 1-1-1.

### Statistical analysis

The statistical analysis was performed with SPSS 27 (IBM, Armonk, New York, USA). Significance tests for group comparison of variables were performed using the chi-squared test. For the other groups, the Kolmogorov-Smirnov was used to evaluate the distribution of the data. Significance tests for data with parametric distribution were performed using an unpaired t-test, and data with a nonparametric distribution were compared with the Mann-Whitney U test. A p-value of ≤ 0.05 was considered to be statistically significant.

## Results

In total 34 thumbs of 32 patients had available data and were included in this trial. Of the initial 42 thumbs, four patients had to be excluded from the study because they could not be reached and another four withdrew their consent to participate. 16 procedures were performed under LA and 18 under BPB.

Baseline results are shown in Table 1.

**Table 1:** Patient collective

Group	LA	BPB	Total	*p*-value
Patients	16	18	32	
Joints	16 (47%)	18 (53%)	34	
Sex				0.289
Women	12 (35%)	16 (47%)	28 (82%)	
Men	4 (12%)	2 (6%)	6 (18%)	
Age				0.836
Mean	54.88	56.44	55.71	
Range	40–67	47–79	40–79	
SD	6.761	9.218	8.074	
Dominant side				0.054
Right	13 (38%)	18 (53%)	31 (91%)	
Left	3 (9%)	0	3 (9%)	
Operated side				0.028
Right	3 (9%)	10 (29%)	13 (38%)	
Left	13 (38%)	8 (24%)	21 (62%)	
Dominant hand operated				0.292
Yes	6 (18%)	10 (29%)	16 (47%)	
No	10 (29%)	8 (24%)	18 (53%)	
^LA: local anaesthesia; BPB: brachial plexus block; SD: standard deviation^				

In both groups, no conversion to another anaesthetic method was required after beginning the initial anaesthetic method. The initial anaesthesia had to be repeated once in each group because the first attempt appeared to be insufficient, as the patients experienced pain. Two patients in the LA group stated afterwards that they would have preferred a different anaesthesia if they were to repeat the operation. The first patient would have wanted another anaesthesia due to failure of the initial LA, and the second patient did not like the feeling of the arm being moved and the noise and action in the operating theatre. Three patients in the BPB-group would have preferred another anaesthesia if they were to repeat the operation. The first patient experienced discomfort during her hospital stay and would have preferred an anaesthetic technique that permitted an outpatient treatment. The other two patients were irritated by the long paralysis of their arm following the BPB.

In the LA group, significantly more patients underwent surgery on their left hand compared to the BPB group (*p* = 0.028). However, there was no significant difference between the two groups regarding whether the dominant hand was operated on (*p* = 0.054).

Results regarding VAS, McGill, and DASH scores are shown in Table 2, and no significant differences were observed between the two groups preoperatively and at the three month follow-up. Additionally, the VAS showed no significant differences between the two groups on the 1^st^ postoperative day and the 12^th^ postoperative day (suture removal).

**Table 2:** VAS, SF-McGill, and DASH scores

Group	LA	BPB	Total	*p*-value
VAS preoperative	6.27	5.76	6	0.501
N	15	17	32	
SD	2.549	2.166	2.328	
VAS 1st day postoperative	3.75	3.25	3.42	0.475
N	8	16	24	
SD	2.55	2.793	2.669	
VAS 12th day postoperative	1.5	1.87	1.74	0.571
N	8	15	23	
SD	1.927	1.685	1.738	
VAS 3 months postoperative	1.8	2.6	2.2	0.397
N	15	15	30	
SD	1.859	2.473	2.188	
SF-McGill preoperative	14.33	17.88	16.22	0.218
N	15	17	32	
SD	8.829	8.092	8.499	
SF-McGill 3 months postoperative	10.27	6.94	8.55	0.321
N	15	16	31	
SD	9.13	6.34	7.865	
DASH score preoperative	55.74	50.85	52.68	0.951
N	9	15	24	
SD	15.98	17.40	16.70	
DASH score 3 months postoperative	30.36	23.34	26.85	0.419
N	15	15	30	
SD	20.28	17.90	19.13	
^*LA*local anaesthesia, *BPB*brachial plexus block*SD*standard deviation, *n*number of patients^				

Results regarding intraoperative pain, intraoperative anxiety, overall satisfaction, sufficiency of pain control, and willingness to reoperate with the same anaesthetic technique are shown in Table 3, with a significant difference observed in intraoperative anxiety (*p* = 0.045).

**Table 3:** Telephone survey

Group		LA (*n* = 16)	BPB (*n* = 18)	Total	*p*-value
Intraoperative pain	Mean	0.56	0.28	0.41	0.483
	SD	1.999	1.179	1.598	
	Range	8 (0–8)	5 (0–5)	8 (0–8)	
Intraoperative anxiety	Mean	1.69	0.28	0.94	0.045
	SD	2.651	0.575	1.969	
	Range	8 (0–8)	2 (0–2)	8 (0–8)	
Overall satisfaction	Mean	9.188	8.944	9.059	0.464
	SD	1.559	1.626	1.575	
	Range	5 (5–10)	5 (5–10)	5 (5–10)	
Postoperative pain medication was sufficient	Yes	12	16	28	0.289
	No	4	2	6	
Willing to reoperate with the same anaesthetic technique	Yes	14	15	29	0.732
	No	2	3	5	
^*LA*local anaesthesia, *BPB*brachial plexus block, *SD*standard deviation, *n*number of patients^					

There was a statistically significant difference noted in operation length (*p* = 0.018) and setup time (*p* < 0.001) when comparing the two groups. The mean operation time in the LA group was 39 min (SD: 7.46), and in the BPB group it was 45 min (SD: 7.02). The mean setup time in the LA group was 76 min (SD: 15.85), and in the BPB group 102 min (SD: 19.66). While the proportion of the operation time in the whole setup time was 44% in the BPB group, it was 51% in the LA group.

## Discussion

The main goal of this study was to determine if LA is an equal alternative to BPB when performing total TMCJ arthroplasty. Statistically significant differences between the two groups investigated were found regarding presence of intraoperative anxiety (*p* = 0.045), operation length (*p* = 0.018), setup time (*p* < 0.001) and operated side (*p* = 0.028).

The patients provided their reasons for higher intraoperative anxiety in the LA group during the telephone survey. They described the feeling of having their hand pulled and that they were too aware of what was happening in the operating theatre. They would have wished to distance themselves more from the ongoing surgery.

When LA was applied, no anaesthesiology team was present, whereas the anaesthesiology team took care of the patient during the whole procedure when BPB was chosen. This could be a confounder of the overall satisfaction and intraoperative anxiety, given that the intraoperative anxiety was significantly higher in the LA group. More effort in providing in-depth information about the whole procedure might be beneficial for the patient experience. Furthermore, patients were able to choose the anaesthetic method, and in this hospital, there is also the option to undergo the operation under general anaesthesia. Therefore, patients who experience higher stress levels during hospital stays may have opted for general anaesthesia initially and were consequently not considered for inclusion in this study. Given the significantly higher intraoperative anxiety and the equal willingness for a hypothetical reoperation, it must be considered that only patients with higher stress tolerance were included and compared.

The results show that the setup time in the BPB group was significantly prolonged compared to the LA group, with a mean of 26 min. This can be explained by the time that the anaesthesiologist needs to perform the axillary block, the safekeeping of the numb limb and the longer operating time. The operation length was also significantly longer in the BPB group than in the LA group, with a mean of 6 min. The surgeon performed the total TMCJ-arthroplasties under LA more recently than those under BPB, therefore the longer operation time in the BPB group might be explained by the learning curve of the surgeon. An operation under BPB is not only an indication for inpatient treatment, but it also takes more time and capacity. From a financial perspective, several studies have shown that outpatient treatment is favourable compared to inpatient treatment [28–32]. With the longer setup time of the BPB, it is to be expected that performing the total TMCJ-arthroplasty under LA offers an opportunity to save resources. The study center where the research was conducted, like many others, is affected by the shortage of specialists [33]. Often, operating theatres and surgical teams are available, but there is no anaesthesiologist to utilize these resources. By performing operations that do not require anaesthesia, personnel capacities could be redistributed and utilized more efficiently.

Regarding the efficiency of BPB, Strub et al. [34] performed a comparison between plexus anaesthesia with and without ultrasound-guidance. In both groups, the musculocutaneous nerve was the most likely to be insufficiently anaesthetized during an axillary block. Given that Cruveilhier’s branch, which arises from the lateral cutaneous nerve of the forearm, also plays a role in the innervation of the TMCJ [26, 27], an inadequate block of the musculocutaneous nerve could result in tourniquet and intraoperative pain when the TMCJ is surgically approached. In this study’s cohort, only one patient in the BPB group experienced an inadequate block that had to be repeated. Due to the small size of the study cohort, it is not possible to determine the influence of Cruveilhier’s branch on the anaesthetic procedure chosen.

One of the main complications of a total TMCJ arthroplasty is the risk of postoperative dislocation [21]. Since the thumb is only numbed but not motor blocked, the surgeon can assess the active range of motion and stability of the prosthesis using a trial neck and head before the implantation of the definitive prosthesis. It can be determined whether impingement occurs or if the prosthesis dislocates at maximum excursions. The tightness of the connective tissue and muscles can also be optimally controlled. Furthermore, the patient experiences no pain during this intraoperative movement and gets a first glimpse of the possible movement directly after the device’s implantation. This could also be motivating for postoperative ergotherapy [14].

To the authors’ knowledge, Müller et al. [13] published the first case report in 2018 on a total TMCJ arthroplasty performed under wide awake local anaesthesia no tourniquet (WALANT). Postulated advantages of performing the procedure under LA are the minimized anaesthetic risk, maximized cost-effectiveness, decreased in-house time, and the possibility of performing an intraoperative assessment of the active stability of the prosthesis. There were no intraoperative incidents concerning the anaesthesia and the postoperative results were satisfactory.

Three years later in 2021, Larsen et al. [12] compared 100 hands of 93 patients in a matched cohort study. Of these, 46 patients (50 hands) received a total TMCJ arthroplasty under WALANT and 47 patients (50 hands) received a total TMCJ arthroplasty under general anaesthesia or regional anaesthesia (RA) in a bloodless field due to an applied tourniquet. Like in this study, no conversions to general anaesthesia or RA were reported after the application of LA. All patients operated on under WALANT were willing to repeat the operation with the same anaesthetic procedure, in contrast to this study, where in both groups (three in the LA group and two in the BPB group) patients would choose another anaesthetic procedure if it were to be repeated. There were no statistically significant differences in the QuickDASH score, grip strength, or the VAS. The mean operation time in the WALANT group was 53 min and 58 min in the bloodless field group. Similar to Larsen et al. similar results are reported in this study, with no significant differences in the DASH and the VAS scores. A longer mean surgery time in the BPB group was also observed, which, as also concluded by Larsen et al., could be attributed to the surgeon’s experience.

In a similar way, Moscato et al. [11] compared 30 hands of 30 patients in a retrospective study. Of these, 15 patients received total TMCJ arthroplasty under WALANT and 15 patients received total TMCJ arthroplasty under local anaesthesia with peripheral nerve blocks (LAPNV). Significant differences between the two groups were found in functional outcomes of the QuickDASH score, with a score of 4.93 in the WALANT group and 13.47 in the LAPNV group (*p* = 0.01) at four months postoperatively, whereas in this study the two groups did not show a significant difference.

Only one study investigated perioperative anxiety in operations for OA of the TMCJ under WALANT, based on a retrospective review of 16 cases, including six total TMCJ arthroplasties [14]. Notably, the anxiety scores differed from those in this study. A score of one indicated no anxiety, whereas in this study, a score of zero indicated no anxiety. Additionally, there was no distinction made between total TMCJ arthroplasties, trapeziectomies, and suture suspension procedures. When their intraoperative anxiety score of 2.25 is converted to this study's scale, it results in an anxiety score of 1.39, which is slightly lower than our intraoperative anxiety score of 1.69 in the LA group. Additionally, there was one reported conversion from WALANT to conventional anaesthesia during a total TMCJ arthroplasty, whereas no conversions occurred in this study's cohort. Naturally, this score conversion and data comparison should be interpreted with caution.

Notably, the anaesthetic techniques used in the previous studies concerning this topic differ slightly from the method used in this study [11–14]. While the studies investigated used WALANT, in this study LA without epinephrine and a tourniquet during the placement of the cup was used. WALANT requires a local anaesthetic mixed with an adrenalin solution to obtain a bloodless operation field [35]. In the hospital where this study was conducted, WALANT is not yet widely used, which is why local anesthesia combined with a tourniquet was applied. Nevertheless, the design of these studies mentioned above are close to this study in methodology. In total joint arthroplasties, periprosthetic joint infections are a concerning complication [36], but consistent data percentages on infections after total TMCJ arthroplasties are lacking [21]. In particular, there is no data on whether WALANT contributes to a higher risk of infection after total TMCJ arthroplasties. However, comparative studies examining WALANT versus conventional anaesthesia in hand surgery did not find a correlation [37, 38]. In the LA and BPB groups of this study, the operation took place under the same sterile conditions in an operating theatre. Nevertheless, the caseload is too small to assess whether LA has a higher risk of postoperative infection compared to BPB.

Regarding safety, patient satisfaction, and willingness to reoperate, the results are in line with other findings reported in the literature [11–14].

### Strengths and limitations

Only two studies have delivered usable data comparing the feasibility of LA in total TMCJ arthroplasty with established anaesthetic methods. This study complements the current level of knowledge with more information and was the first to compare intraoperative anxiety and sufficiency of basic pain medication in total TMCJ arthroplasty under LA with BPB.

The limitations of this study include the small sample size, the lack of randomization, the retrospective design and the extended follow-up period of the telephone survey. Generally, patients could choose whether they wanted LA or BPB. While all patients in the LA group had this choice, patients in the BPB group who were operated on before the introduction of LA could only choose between BPB and general anaesthesia. Another limitation was incomplete questionnaire follow-up.

## Conclusion

In this non-randomized cohort study, LA and BPB in total TMCJ arthroplasty were compared. The results indicate that LA is equivalent to BPB in terms of functional outcome, pain reduction, and willingness to repeat the procedure. This study also shows a higher intraoperative anxiety when LA was applied. This approach benefits from preserving anaesthesiology resources and reducing overall costs. It is recommend to actively involve the patient in the selection of the anaesthetic method to achieve the most satisfactory outcome.
